# Cardiac troponin at the point of care in acute and chronic coronary syndromes

**DOI:** 10.1111/dom.16503

**Published:** 2025-06-16

**Authors:** Michael McDermott, Yong Yong Tew, Jamie G. Cooper, Deepak Harry, Nicholas L. Mills

**Affiliations:** ^1^ BHF Centre for Cardiovascular Science University of Edinburgh Edinburgh UK; ^2^ Aberdeen Royal Infirmary Aberdeen UK; ^3^ Royal Infirmary of Edinburgh Edinburgh UK; ^4^ Usher Institute University of Edinburgh Edinburgh UK

**Keywords:** cardiovascular disease, macrovascular disease, real‐world evidence, systematic review

## Abstract

High‐sensitivity cardiac troponin (hs‐cTn) assays are integral to the assessment of patients with acute chest pain where prompt and accurate diagnosis of myocardial infarction enables timely delivery of potentially life‐saving treatment. In the Emergency Department, implementation of central laboratory hs‐cTn assays has reduced length of stay and unnecessary hospital admission. Recently hs‐cTn assays have become available on point‐of‐care (POC) platforms that have equivalent analytical and diagnostic performance to central laboratory platforms but can deliver results more rapidly to guide decisions in real‐time. These assays have the potential to further accelerate care in the Emergency Department and to facilitate the assessment of patients with suspected acute coronary syndromes in pre‐hospital settings, primary care and the out‐patient clinic. Greater accessibility to testing could broaden the role of hs‐cTn to improve the risk stratification of patients with new onset exertional angina and chronic coronary syndromes. Whilst hs‐cTn assays are already available for use at the point‐of‐care, prospective studies are required to determine the clinical and cost‐effectiveness of testing in patients with acute and chronic coronary syndromes.

## 1 INTRODUCTION

Cardiovascular disease remains the leading cause of morbidity and mortality, resulting in substantial loss of economic productivity and accounting for 1 in 7 deaths.^1^, ^2^, ^3^ Patients with co‐existing diabetes mellitus and cardio‐renal disease are at particular risk of cardiovascular events, including acute coronary syndromes,^4^ and those with diabetes mellitus often have more advanced coronary artery disease and a worse prognosis, compared to those without.^5^

In the last year alone, there were 15 million presentations to an Emergency Department with chest pain in Europe and the United States, accounting for approximately 1 in 10 attendances and 4 in 10 unscheduled admissions to the hospital.^6^, ^7^, ^8^ The evaluation of chest pain places a significant burden on healthcare providers, contributes to overcrowding in the Emergency Department, and is associated with worse outcomes for all patients in this setting, irrespective of their presenting complaint.^9^, ^10^ Therefore, care pathways that accelerate diagnosis and allow alternative settings for assessment outside of an Emergency Department environment are a clinical priority.

The introduction of high‐sensitivity cardiac troponin (hs‐cTn) assays on central laboratory platforms has been instrumental to the development of accelerated diagnostic pathways in the Emergency Department.^11^, ^12^, ^13^ For those without myocardial infarction, single‐sample rule‐out pathways can avoid unnecessary serial cardiac troponin measurements or further diagnostic testing, reducing length of stay in the Emergency Department.^14^ For those with myocardial infarction, the time to diagnosis and treatment is reduced. High‐sensitivity assays on central laboratory platforms have also improved the assessment and risk stratification of patients in other clinical settings.^15^, ^16^, ^17^

Recent advances in technology have resulted in point‐of‐care (POC) platforms that can measure cardiac troponin with equivalent analytical performance to those high‐sensitivity assays available on a central laboratory platform.^18^, ^19^, ^20^ The ability to measure cardiac troponin in settings remote from central laboratories, reduce turnaround times and avoid the need for venepuncture through the use of capillary samples is a major step forward in diagnostic testing. This review identifies where hs‐cTn at the point of care has the greatest potential to impact the assessment of patients with acute or chronic coronary syndromes and summarises the current evidence and requirements for prospective studies.

## 2 ACUTE AND CHRONIC CORONARY SYNDROMES

Acute coronary syndromes encompass a spectrum of acute conditions, including ST‐elevated myocardial infarction (STEMI), non‐ST‐elevated myocardial infarction (NSTEMI) and unstable angina.^21^ These presentations have a common underlying pathophysiology, with the majority due to coronary atherosclerotic plaque rupture with thrombosis resulting in myocardial ischaemia. The mortality of acute coronary syndromes is highest within hours of symptom onset, and the prompt diagnosis can allow timely delivery of life‐saving treatments that improve prognosis.^22^ The electrocardiogram is essential for differentiating STEMI from NSTEMI, whilst measuring cardiac troponin is required to rule in or rule out myocardial infarction in those without ST‐segment elevation and to differentiate NSTEMI from unstable angina. For a diagnosis of myocardial infarction, a rise and/or fall of cardiac troponin is required, with at least one value above the 99th percentile upper reference limit (URL), in addition to one of the following: symptoms of myocardial ischaemia, new ischaemic changes on the electrocardiogram, the development of pathological Q‐waves, imaging evidence of new loss of viable myocardium or new regional wall motion abnormality, or identification of atherothrombosis on coronary angiography.^23^

The term chronic coronary syndrome is more recent and encompasses a range of clinical presentations due to diseases of the coronary arteries and/or microcirculation resulting in structural or functional abnormalities that result in transient myocardial ischaemia.^24^ At the macrovascular level, these include focal or diffuse atheroma, myocardial bridging, congenital arterial anomalies or transient coronary artery vasospasm, whilst coronary microvascular dysfunction may be responsible at the microvascular level.^24^ These may result in reversible myocardial ischaemia that arises with exertion and may manifest as angina, dyspnoea or be asymptomatic. Although chronic coronary diseases can be stable, they can also be progressive and dynamic with episodes of acute chest pain requiring expedited assessment. Whilst measuring cardiac troponin is recommended by clinical practice guidelines to exclude acute myocardial infarction, hs‐cTn assays may also provide insights into the risk of future adverse cardiovascular events in those with chronic coronary syndromes.^16^, ^25^, ^26^

## 3 HIGH‐SENSITIVITY CARDIAC TROPONIN AND POINT‐OF‐CARE ASSAYS

**FIGURE 1:**
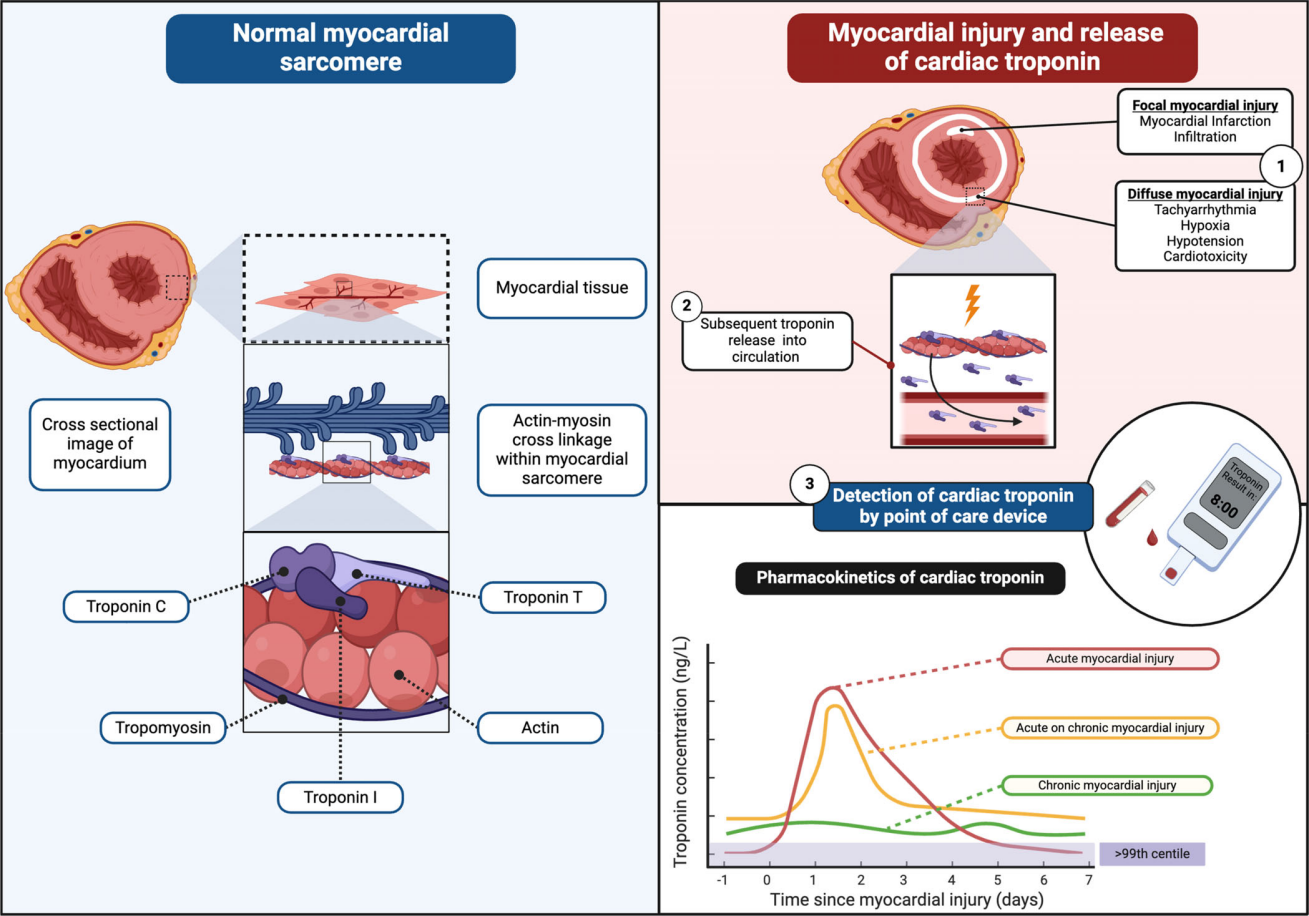
Overview of normal sarcomere and release of cardiac troponin following acute myocardial injury.

Cardiac troponins are key regulatory proteins found upon the thin filament of the myocardial sarcomere and are released into the circulation following myocardial injury or necrosis (Figure 1). Unlike biomarkers previously used in the diagnosis of myocardial infarction, cardiac troponins are highly specific for myocardial injury.^27^ Acute myocardial injury is defined as a rise and/or fall in cardiac troponin with at least one value above the assay‐specific 99th percentile URL. As cardiac troponin concentrations differ in males and females,^28^ sex‐specific 99th percentile URLs are recommended for all assays.^23^ Cardiac troponin concentrations above the assay‐specific 99th percentile URL with a less than 20% change on serial measurements are used to define those with chronic myocardial injury. Chronic myocardial injury is associated with adverse cardiovascular outcomes and arises as a consequence of coronary artery disease, structural heart disease or chronic kidney disease, amongst other conditions.^29^

High‐sensitivity assays are now able to accurately and precisely quantify very low levels of cardiac troponin even in healthy persons, allowing earlier detection of changes in cardiac troponin concentrations. The analytical precision of an assay is determined by the assay coefficient of variation in replicate measurements as the standard deviation divided by the mean concentration expressed as a percentage. The International Federation of Clinical Chemistry (IFCC) defines a high‐sensitivity assay as one that has a coefficient of variation ≤10% at the sex‐specific 99th centile URL,^30^, ^31^ and can measure cardiac troponin above the assay limit of detection in more than half of healthy females and males within a reference population.^30^, ^31^, ^32^ Until recently, only central laboratories were able to achieve these requirements, and samples had to be processed by trained laboratory technicians, with recommended turnaround times of around 1 h from sampling to reporting.^31^, ^33^ This is often not met as the time to transfer specimens to the laboratory is often longer, even in secondary or tertiary hospitals where the central laboratory is on site. Furthermore, there are often delays in actioning the results in the electronic patient record, even when they are reported efficiently.

Point‐of‐care devices measuring cardiac troponin have been available for many years. These assays are often referred to as contemporary assays, but for the purpose of this review, they are classified as not high‐sensitivity. These assays have high specificity for the diagnosis of myocardial infarction but low sensitivity and poor precision at low cardiac troponin concentrations.^32^ It is therefore not possible with these assays to accurately define the 99th percentile URL, and instead, higher thresholds based on analytical precision have been used. However, in recent years, new point‐of‐care platforms have been introduced that do satisfy the IFCC requirements for an hs‐cTn assay (Table 1).^32^, ^34^ The ability to measure cardiac troponin by non‐laboratory personnel at the point of care in venous or capillary whole blood has the potential to transform the use of high‐sensitivity assays by enabling real‐time testing and risk assessment in a broader range of clinical settings.

**TABLE 1:** Summary of point of care cardiac troponin I and T assays analytical characteristics.

Company	Platform	Assay	Time to result	Specimen type	LoB	LoD	Percent normals measured ≥ LoD	99th percentile	% CV at 99th percentile	Concentration at 10% CV
High sensitivity assays										
LSI Medience (formerly Mitsubishi)	PATHFAST	hs‐cTnI/cTnI‐II	17 min	Heparin‐Na, heparin‐Li or EDTA whole blood or plasma	1.23 ng/L	2.33 ng/L	Overall: 66.3% F: 52.8% M: 78.8%	Overall: 27.9 ng/L Female: 20.3 ng/L Male: 29.7 ng/L	6.1%	15 ng/L
Quidel/Alere	TriageTrue	hs‐cTnI	<20 min	EDTA whole blood or plasma	0.4 ng/L (plasma) 0.5–0.8 ng/L (whole blood)	0.7–1.6 ng/L (plasma) 1.5–1.9 ng/L (whole blood)	Overall: ≥50%	Overall: 20.5 ng/L Female: 14.4 ng/L Male: 25.7 ng/L	5.0–5.9% at 21 ng/L (plasma) 5.9–6.5% at 22 ng/L (whole blood)	4.4–8.4 ng/L (plasma) 5.8–6.2 ng/L (whole blood)
Siemens	Atellica VTLi	hs‐cTnI	8 min	Li Hep whole blood and plasma, capillary blood	0.55 ng/L	1.2 ng/L (plasma) 1.6 ng/L (whole blood)	Overall: 83.7% F: 79.7% M: 87.3%	Overall: 23 ng/L Female: 18 ng/L Male: 27 ng/L	6.5% (plasma) 6.1% (whole blood) at 22.9 ng/L	6.7 ng/L (plasma) 8.9 ng/L (whole blood)
SpinChip diagnostics	SPINCHIP	hs‐cTnI	10 min	Li Hep whole blood and plasma, capillary blood	Not available	1.1 ng/L (plasma) 1.2 ng/L (whole blood)	Overall: 75% Female: 62% Male: 88%	Preliminary results: Overall: 31.7 ng/L Female: 27.3 ng/L Male: 36.9 ng/L	4.5% (plasma) 4.2% (whole blood) at 31.7 ng/L	Not available
Non‐high sensitivity assays										
Abbott	i‐STAT	cTnI	10 min	Na and Li heparinised whole blood and plasma	0.02 μg/L	Not available	Not available	Overall: 0.08 μg/L	16.5%	0.1 μg/L
Radiometer	AQT90 FLEX	TnI	<19 min	EDTA and heparinised whole blood and plasma	<0.005 μg/L	<0.010 μg/L	Not available	Overall: 0.023 μg/L	12.9%	0.027 μg/L
Radiometer	AQT90 FLEX	TnT	12 min	EDTA and heparinised whole blood and plasma	0.005 μg/L	0.010 μg/L	Not available	Overall: 0.017 μg/L	15.2%	0.026 μg/L
Response biomedical	RAMP	Troponin I	15 min	Only EDTA whole blood	Not available	0.03 μg/L	Not available	Overall: <0.10 μg/L	20.0%	0.21 μg/L
Roche	Cobas h 232	Cardiac POC troponin T	12 min	Heparinised whole blood	Not available	0.04 μg/L	Not available	Not available	Not available	9.3% between 0.04 and 0.2 μg/L
Siemens	STRATUS CS Acute Care	cTnI	14 min	Whole blood (Li or Na heparin) or plasma Li or Na heparin	<0.03 μg/L	Not available	Not available	Overall: 0.07 μg/L	8.2%	0.06 μg/L

Abbreviations: CV, coefficient of variation; EDTA, ethylenediaminetetraacetic acid; hs‐cTnI, high‐sensitivity cardiac troponin I; Li, lithium; LoB, limit of blank; LoD, limit of detection; Na, sodium; POC, point of care; TnI, troponin I; TnT, troponin T.

*Source*: Adapted from the International Federation of Clinical Chemistry and Laboratory Medicine–Clinical Applications of Cardiac Bio‐Markers Updated tables (https://ifccfiles.com/2024/03/Point‐of‐Care‐Cardiac‐Troponin‐I‐and‐T‐Assay‐Analytical‐Characteristics‐Designated‐By‐Manufacturer‐v062024.pdf).

## 4 OPPORTUNITIES FOR HIGH‐SENSITIVITY CARDIAC TROPONIN AT THE POINT OF CARE

### 4.1 Primary care and community treatment centres

Measuring cardiac troponin using central laboratory hs‐cTn assays has not been practical within primary care or outpatient settings, due to geographical distance from central laboratory sites and unacceptable turnaround times from sampling to reporting. Point‐of‐care hs‐cTn testing now allows the rapid measurement in settings without central laboratories.

Whilst many patients with chest pain at rest or new onset exertional chest pain attend the Emergency Department, chest pain still accounts for 1%–3% of all appointments in primary care.^35^ In urban areas, patients with possible acute coronary syndromes are often redirected to secondary care facilities for assessment. In remote and rural communities, however, this is often impractical, where patient transfer times can be long and costly. This is especially pertinent given that in some studies, as few as 4% of patients attending the hospital with chest pain have a final diagnosis of myocardial infarction.^36^, ^37^ Excluding myocardial infarction in primary care using an hs‐cTn assay at the point of care could negate the need for transfer to secondary care facilities and reduce overcrowding in the Emergency Department. Conversely, for those found to have myocardial injury in this setting, earlier use of antiplatelet agents and direct transfer to tertiary cardiac centres could plausibly improve outcomes.

Exclusion of myocardial infarction using assays that are not high‐sensitivity with point‐of‐care devices has required serial testing, due to the low diagnostic sensitivity of these assays and the potential to miss myocardial infarction with a single sample.^38^, ^39^ Small prospective studies using non‐high‐sensitivity assays within primary care have demonstrated fewer patients were referred to hospital for assessment when point‐ofof‐care testing was introduced.^39^ This, however, was at the expense of missing a small number of patients with acute myocardial infarction or unstable angina, who would have been detected with hs‐cTn assays.^38^ Further studies have demonstrated the benefit of adapting accelerated diagnostic chest pain pathways for non‐high‐sensitivity cardiac troponin at the point of care in rural New Zealand.^40^, ^41^ The use of an electrocardiogram, risk score and a non‐high‐sensitivity cardiac troponin assay identified 44% of patients as low risk.^40^ This allowed the majority of low‐risk patients to avoid transfer to emergency care centres, with no major adverse cardiovascular events at 30 days.^40^

The availability of hs‐cTn assays at the point of care could allow the single‐sample rule‐out of myocardial infarction in primary care, without serial testing.^42^ However, it is important to highlight that some patients will require serial measurements if their symptoms occurred within 3 hours of sampling or the cardiac troponin concentration is above the rule‐out threshold. In a single‐centre observational cohort study, hs‐cTn testing in primary care using a central laboratory assay identified 76.6% of patients as low risk with a sensitivity and negative predictive value (NPV) of 98.4% and 99.9%, respectively.^43^ The safety and efficacy of measuring cardiac troponin at the point of care with a high‐sensitivity assay in primary care will be evaluated in the randomized controlled trial POB‐HELP study (*Ruling out acute coronary syndrome in primary care with a clinical decision rule and a capillary, high‐sensitive troponin I point of care test; NCT05827237*).^44^

Availability of hs‐cTn at the point of care in a primary care setting offers opportunities beyond the rule‐in and rule‐out of myocardial infarction. International guidelines for the primary prevention of cardiovascular disease currently recommend the use of population‐based cardiovascular risk scoring.^45^, ^46^ These risk scores, however, have been shown to overpredict cardiovascular risk in older adults and underestimate risk in younger individuals. Large studies have demonstrated that incorporating cardiac troponin into risk prediction models improves the accuracy of risk scores.^26^, ^47^, ^48^ Higher concentrations are associated with more cardiovascular events and deaths compared to those with lower concentrations.^47^, ^48^ Interestingly, cardiac troponin testing in asymptomatic patients with diabetes mellitus demonstrates a link between glucose control, troponin concentration and future events.^49^ In the future, having access to cardiac troponin results in real time could help in decision‐making and inform the use of primary preventative therapies.

Patients may also present to primary care with new exertional symptoms suggestive of stable angina. At present, there is little access to objective diagnostic testing within primary care, which makes selecting patients for emergency care and expedited outpatient review challenging.^50^, ^51^ Access to hs‐cTn testing at the point‐of‐care could support decision making in primary care by excluding an acute coronary syndrome as the cause of new onset exertional angina and providing an objective measure of risk to identify those patients who are more likely to benefit from expedited assessment at specialist outpatient clinics. The feasibility of this approach is currently being explored in the ORACLE Heart study (*Objective Risk Assessment in Patients with Possible Anginal Chest Pain using Leading Technology; NCT 06325020*).

### 4.2 Specialist outpatient clinics

High‐sensitivity cardiac troponin testing is not currently used routinely in the assessment of patients with chronic coronary syndromes or structural heart disease, as the results from a central laboratory are not available in real time. The development of hs‐cTn POC assays could support new applications for testing in this setting, including the risk stratification and optimisation of medical therapies in patients reviewed in the outpatient clinic following an acute coronary syndrome and in the evaluation of patients with possible new‐onset angina.

#### 4.2.1 Risk stratification following an acute coronary syndrome

The term *chronic coronary syndrome* includes patients with a stabilised acute coronary syndrome or following coronary artery revascularisation.^24^, ^52^ The clinical trajectory and prognosis of patients in this setting can be unpredictable, with not all patients at equal risk of future events.^21^, ^24^, ^53^

At present, international guidelines recommend using clinical criteria to help identify those at greatest risk of future cardiovascular events, which include those with more than one previous major cardiovascular event, diabetes mellitus, chronic kidney disease, age and persistently elevated low‐density lipoprotein cholesterol or an unfavourable anatomical distribution of coronary artery disease.^24^, ^53^

In those identified at the greatest risk, targets for low‐density lipoprotein cholesterol are lower, dual anti‐platelet therapies are administered for longer, or the co‐administration of anti‐coagulant therapies is considered to further reduce the recurrence of cardiovascular events.^21^, ^24^, ^54^, ^55^ Personalized risk stratification with cardiac troponin may improve patient selection for additional therapies. The potential utility of cardiac troponin as a risk stratification tool in this setting has been demonstrated in multiple observational studies where those with higher cardiac troponin concentrations are at increased risk and testing out performs existing risk prediction tools^56^, ^57^, ^58^, ^59^ Furthermore, in patients with type 2 diabetes mellitus, high sensitivity cardiac troponin has been shown to predict adverse cardiovascular outcomes independently of diabetes severity, cardiovascular risk factors and coronary anatomy.^60^ However, at present no studies have evaluated the impact of implementing hs‐cTn testing in this setting using either a central laboratory or POC assay.

In the PROVE‐IT‐TIMI 22 study, patients with higher cardiac troponin concentrations measured using a central laboratory hs‐cTn assay at least 30 days post myocardial infarction had a higher incidence of cardiovascular death or heart failure hospitalization compared to those with lower concentrations (6.2% vs. 1.7%).^61^ Furthermore, the absolute risk reduction observed in patients randomized to high‐intensity statins was greater in those with higher cardiac troponin concentrations relative to those with lower levels (absolute risk reduction 3.5% vs. 0.4%).^61^ In the EXAMINE trial (Examination of Cardiovascular Outcomes with Alogliptin versus standard of care), patients with type 2 diabetes mellitus who were more than 30 days post hospitalization for acute coronary syndrome, with higher cardiac troponin concentrations on a central laboratory hs‐cTn assay were at greater risk of cardiovascular death, non‐fatal myocardial infarction and non‐fatal stroke at 2 years relative to those with concentrations below the assay's limit of detection (22.6% vs. 4.1%).^62^ Furthermore, when patients were risk stratified by cardiac troponin alone, it more accurately identified those at risk of future cardiovascular events compared with the current guideline‐recommended strategies.^53^, ^58^ Serial hs‐cTn testing with a central laboratory assay in the 12 months following myocardial infarction suggests that both the absolute troponin concentration and trajectory are independent predictors of future cardiovascular death.^63^

To date these observations have been interesting but based on retrospective analyses in stored material. Furthermore, it has not been practical to implement central laboratory hs‐cTn testing in the clinic because of the long turnaround times. The availability of hs‐cTn POC testing makes implementation possible and will support prospective studies to determine whether risk stratification with cardiac troponin testing following myocardial infarction influences the use of secondary prevention and improves outcomes.

#### 4.2.2 Risk stratification in patients with possible new onset angina

For patients referred to specialist cardiac services with possible new onset angina, guidelines currently recommend calculating the pre‐test probability that symptoms are attributable to coronary artery disease to guide further investigations.^24^, ^52^ These prediction models, however, have been shown to incorrectly estimate the likelihood of coronary artery disease, highlighting the requirement for alternative approaches to improve patient selection.^50^, ^64^

In patients with possible angina undergoing coronary CT angiography, cardiac troponin measured in stored material using a hs‐cTn assay in a central laboratory correlated with both the severity of coronary artery disease on coronary CT angiography and the risk of future cardiovascular events.^65^, ^66^, ^67^ This was also observed in patients with probable stable angina undergoing invasive coronary angiography where those with cardiac troponin concentrations >10 ng/L on a central laboratory hs‐cTn assay had a greater incidence of future non‐fatal myocardial infarction or cardiovascular death compared to those below this threshold.^25^ Furthermore, in those with cardiac troponin concentration >10 ng/L who underwent revascularisation, there was an association with lower risk of future non‐fatal myocardial infarction or cardiovascular death that was not observed in those undergoing revascularisation with values below this threshold.^25^

### 4.3 Emergency and acute care

Chest pain remains one of the most common presentations to the Emergency Department; however, only a small proportion of patients have myocardial infarction.^36^, ^37^ Multiple accelerated diagnostic pathways that are based on hs‐cTn assays from central laboratories have now been endorsed by clinical practice guidelines.^21^, ^68^, ^69^ Single‐sample rule‐out pathways identify between 20% and 50% of patients as low risk of myocardial infarction in the Emergency Department, and in randomised controlled trials, implementation reduced the need for serial testing and hospital admission.^11^, ^17^, ^70^ Single‐sample rule‐out pathways rely on risk stratification thresholds that can only be measured using a high‐sensitivity assay, close to the limit of detection.

However, the clinical effectiveness of single‐sample rule‐out pathways is diminished by lengthy turnaround times due to sample transportation, processing, analysis and reporting by central laboratories.^71^, ^72^, ^73^ Measuring cardiac troponin with a high‐sensitivity assay at the point of care could circumvent many of these delays.^74^, ^75^, ^76^

Whilst single‐sample rule‐out pathways were initially developed using central laboratories, several observational cohort studies have now demonstrated diagnostic performance equivalent to central laboratory assays using several hs‐cTn assays on point‐of‐care devices. A single‐sample rule‐out threshold for the Atellica VTLi hs‐cTnI assay was derived using whole blood (Safe Emergency Department Discharge Rate, NCT04772157; *n* = 1086 patients; incidence of myocardial infarction 8.1%) and subsequently validated using stored plasma (Suspected Acute Myocardial Infarction in Emergency, ACTRN12621000053820; *n* = 1486 patients; incidence of myocardial infarction 5.5%). A cardiac troponin I concentration of <4 ng/L could identify 40% of patients as safe for discharge home with a single sample, for a sensitivity of 98.8% (95% confidence interval [CI], 93.3%–100%) and negative predictive value (NPV) of 99.8% (95% CI, 99.1%–100%) for myocardial infarction.^77^ Adverse cardiac events at 30 days occurred in 0.1% (*n* = 1) and 0.8% (*n* = 5) in those below this threshold in the derivation and validation cohorts, respectively.^77^ Similar findings were observed with the TriageTrue high‐sensitivity cardiac troponin I assay, which identified 45% of patients as low risk of myocardial infarction, with a rule‐out threshold of <3 ng/L using stored plasma (APACE [Advantageous Predictors of Acute Coronary Syndromes Evaluation Study], NCT00470587, *n* = 1261 (internal validation cohort, *n* = 545); incidence of myocardial infarction: 14%) with a sensitivity and NPV of 100%.^78^ Other studies have also demonstrated similar safety and efficacy using other cardiac troponin assays available at the point of care.^20^, ^79^

The WESTCOR‐POC study (NCT05354804; *n* = 1494 patients) is the first randomised trial evaluating the implementation of a hs‐cTn POC assay within the Emergency Department, comparing a 0/1‐hour serial testing pathway using central laboratory testing with a similar pathway using point of care testing.^80^, ^81^ The trial met the primary outcome of reducing the median length of stay in the Emergency Department; however, the reduction of 6 min (180 vs. 174 min) is unlikely to persuade other sites to adopt point of care testing.^80^, ^81^, ^82^ The small change in length of stay may be explained by the efficiency of the central laboratory and the study design. All patients underwent serial sampling at 0 and 1 hour with no option to exclude myocardial infarction using a single sample, which may have hampered the potential of point of care testing to accelerate decision making.

A large‐scale evaluation of the effectiveness and safety of implementing a hs‐cTn POC assay in the Emergency Department is underway using a stepped‐wedge cluster randomised design in New Zealand (ICare‐FASTER, ACTRN12619001189112). Preliminary reports from this study suggest that when compared to central laboratory testing, the implementation of hs‐cTn testing at the point of care within a single‐sample rule‐out pathway reduced length of stay in the Emergency Department by approximately 32 min.^83^, ^84^ Whether these findings are replicable across multiple centres and whether further reductions in the length of stay in the Emergency Department are safe is not yet known.

The main reason for using a hs‐cTn POC assay in the Emergency Department is to reduce the turnaround time from blood sampling to reporting. It is unlikely that simply substituting a central laboratory assay with a point‐of‐care test without modification to the diagnostic pathway is going to result in substantial changes in length of stay. The impact of hs‐cTn POC may be minimal in patients where other investigations that are only available in the central laboratory are needed to exclude alternative diagnoses. Some point‐of‐care platforms can support additional measurements such as haemoglobin, glucose, electrolytes, D‐dimer and lactate, which may reduce requirements for central laboratory testing. Furthermore, the clinician's (dis)comfort in making rapid decisions based upon a single cardiac troponin on a POC platform without a period of observation may be a challenge. In the future, integrating results from point‐of‐care testing into machine learning algorithms could support clinical decision making to optimize accelerated diagnosis and care.^85^, ^86^

### 4.4 Ambulance and emergency medical services

Patients with chest pain often seek assistance by calling emergency medical services.^87^, ^88^, ^89^ Pre‐hospital pathways are now established that allow direct transfer to cardiac centres for coronary angiography and prompt reperfusion in patients with ST‐segment elevation on the electrocardiogram without further testing.^90^ These pre‐hospital pathways have demonstrated a reduction in mortality,^91^, ^92^ and are recommended by international guidelines.^21^, ^93^

In most cases, however, the electrocardiogram does not identify ST‐segment elevation,^91^ and therefore patients are transferred to the nearest hospital for further assessment, and not necessarily to specialist cardiac centres. Measuring cardiac troponin at the point of care could avoid transfer to the hospital or facilitate pre‐hospital triage. Those with an elevated cardiac troponin could be directly transferred to cardiac centres, facilitating further assessment and timely revascularisation where needed. Whilst a single low cardiac troponin concentration measured using an hs‐cTn POC assay may exclude myocardial infarction, serial testing is required in those undergoing assessment within 3 hours of symptom onset. The lag in the release of cardiac troponin from the myocardium, following the onset of myocardial infarction, must be recognised to avoid incorrectly excluding myocardial infarction in patients presenting within a few hours of symptom onset. Furthermore, even if myocardial infarction is correctly excluded, other potentially life‐threatening conditions may still require conveyance to the hospital for further investigation, including acute aortic syndromes and pulmonary embolism.

To date, most studies measuring cardiac troponin at the point of care in the pre‐hospital setting have used assays that are not high‐sensitivity. These have been shown to aid in the triage of those patients without ST‐segment elevation on the electrocardiogram,^94^, ^95^, ^96^, ^97^, ^98^, ^99^, ^100^ reducing secondary ambulance transfers, time to coronary angiography, and length of hospital stay, without improving mortality.^101^, ^102^, ^103^ Whilst these pathways have high specificity for myocardial infarction, the assays lack the sensitivity to safely exclude myocardial infarction.^94^, ^95^, ^96^, ^97^, ^98^, ^99^ With growing pressures on all health‐care services, the ability to safely exclude myocardial infarction in a pre‐hospital setting could avoid unnecessary hospital attendances, reduce overcrowding and save costs to healthcare providers.^9^, ^104^

Using risk scores in combination with point‐of‐care testing in the pre‐hospital setting may help identify those who are at high or low risk and could have myocardial infarction ruled in or out, respectively.^105^, ^106^, ^107^, ^108^, ^109^ For those identified as low risk, however, this strategy has not met recognised safety thresholds that can be achieved by care pathways in the Emergency Department.^107^, ^110^ Whilst pre‐hospital testing may not avoid hospital transfer, it may facilitate alternative pathways of care for these low‐risk patients or could form the first of a two‐measurement strategy to be completed in the Emergency Department.^111^ For those identified as high‐risk of myocardial infarction, the use of risk scores had some evidence of benefit, but did not provide additional discrimination over a pre‐hospital cardiac troponin measurement above the URL.^103^, ^107^, ^108^, ^110^, ^112^, ^113^

There have been two studies, both testing cardiac troponin at the point of care using assays that are not high‐sensitivity, in which patients identified as low risk were not routinely transferred to the hospital. The FamouS Triage study enrolled 700 and 536 patients over two phases, where in the second phase, patients identified as low risk by cardiac troponin were not transferred to hospital.^114^ Whilst no differences in myocardial infarction, urgent revascularisation or all‐cause death were observed at 45 days, 1 in 4 low‐risk patients subsequently attended the Emergency Department.^107^ The ARTICA trial randomised low‐risk patients with suspected myocardial infarction without ST‐segment elevation to cardiac troponin testing along with HEAR scoring or standard care.^104^ The study demonstrated significant cost savings in those randomized to point of care cardiac troponin testing. Whilst not powered to assess safety, 2/419 (0.5%) patients triaged as low risk had a myocardial infarction within 30 days, compared with 4/417 (1%) in the standard care arm.^104^ No difference in adverse cardiovascular outcomes was observed between the two strategies at 1 year.^115^

To date, there have been no prospective studies evaluating high‐sensitivity cardiac troponin assays at the point of care solely in a pre‐hospital setting. Data from retrospective studies that have incorporated cardiac troponin measurements from a central laboratory hs‐cTn assay in stored pre‐hospital samples would suggest that 20–30% of patients could be identified as low risk; however, further data on the safety of this approach is needed prior to implementation.^110^, ^116^, ^117^ The lag time from the onset of myocardial infarction to a rise in cardiac troponin must be considered in this group of pre‐hospital patients, where a higher proportion are more likely to have early medical contact (<3 h) compared to those who present to the Emergency Department and may be more likely to require serial testing.

## 5 CHALLENGES OF IMPLEMENTING POINT OF CARE TESTING

The successful integration of hs‐cTn testing at the point of care requires input from multiple stakeholders. There cannot simply be a substitution of central laboratory with point of care testing without careful consideration of the setting, diagnostic pathway, the experience of the clinicians, type of assay and cost to the service.

Clinicians must undergo training and education on the interpretation of results, particularly in special circumstances, such as patients presenting soon after the onset of symptoms (within 3 h), those with renal disease, and those with potential alternative causes of acute or chronic myocardial injury.

In most clinical settings, point of care devices will be used by clinical staff and education must be provided on how best to minimize pre‐analytical variation, particularly with capillary sampling to avoid clotting of samples or overloading of cartridges. Regular device maintenance, calibration, performance monitoring and quality control are essential to maintain the reliability of the device.^118^, ^119^ These points are particularly pertinent to pre‐hospital settings where the user environment can be at extremes of temperatures, remote from power sources/charging points, storage of cartridges at ambient temperatures is difficult, and when testing may be conducted in a moving vehicle.

Successful integration of point of care testing also requires connectivity to the laboratory and electronic patient record to allow reporting and recording of results for clinical governance purposes. If serial testing is required, the lack of harmonisation between the point of care and central laboratory assays must be recognised and acknowledged by all clinical staff. If different assays are used within the same hospital system, each assay must be clearly labelled and reported with the assay‐specific reference range.

Discordance between hs‐cTn assays on central laboratory assays is well recognised, as there has been no attempt to standardise assays.^120^ Differences are particularly marked when comparing cardiac troponin values using a cTnI and cTnT assay.^120^ If serial measurements are required in the same setting, testing should be performed on the same assay, as otherwise, changes in cardiac troponin concentration will not be reliable. If the patient is being transferred from a setting that uses a hs‐cTn POC assay to one that uses a hs‐cTn assay in the central laboratory, then surplus sample from the initial sample may need to be retained to establish the baseline value on the central laboratory assay.

## 6 LIMITATIONS OF THE EVIDENCE AND NEED FOR RESEARCH

Given hs‐cTn assays available at the point of care have similar analytical precision to central laboratory hs‐cTn assays, once cleared by regulatory bodies they can be used for the diagnosis of myocardial infarction in practice. However, users should be aware that most studies evaluating the diagnostic performance of hs‐cTn POC assays have been retrospective, with testing performed in stored plasma rather than whole blood. Prospective studies in whole blood are needed to confirm diagnostic performance and to establish optimal thresholds for use in accelerated diagnostic pathways. Furthermore, whilst hs‐cTn POC assays should reduce the time to clinical decision‐making, other factors, such as the availability of clinicians to review patients in the Emergency Department and the efficiency of the local central laboratory, are likely to influence the impact of point of care testing. To understand the clinical and cost effectiveness of hs‐cTn POC assays in this setting requires implementation studies and randomised trials. Use of point‐of‐care platforms extending hs‐cTn testing to pre‐hospital settings, primary care and out‐patient clinics is likely to have a greater impact on patient care. However, the evidence base to support use in these areas is currently founded entirely on observational studies using central laboratory assays. Before implementing hs‐cTn POC assays in these areas, prospective studies are essential to understand both safety and efficacy and how testing influences care.

## 7 CONCLUSIONS

High‐sensitivity cardiac troponin assays matching the sensitivity of central laboratories are now available at the point of care and offer opportunities to develop new diagnostic care pathways for patients with acute and chronic coronary syndromes. At present, evidence for point of care testing in clinical practice is limited, but multiple prospective and randomised studies are underway that will inform how to safely and effectively integrate testing at the point of care into practice across multiple settings.

## CONFLICT OF INTEREST STATEMENT

NLM has received honoraria or consultancy from Abbott Diagnostics, Roche Diagnostics, and Siemens Healthineers within the last 36 months. All other authors have no interests to declare.

## PEER REVIEW

The peer review history for this article is available at https://www.webofscience.com/api/gateway/wos/peer-review/10.1111/dom.16503.

## Data Availability

Data sharing not applicable to this article as no datasets were generated or analysed during the current study.
